# Navigated robotic-arm-assisted hybrid multi-portal endoscopic fenestration of a thoracic intradural arachnoid cyst: a technical case report

**DOI:** 10.1093/jscr/rjag998

**Published:** 2026-11-04

**Authors:** Cecilia Wanchen Huo, Ralph J Mobbs, Yi Yuen Wang

**Affiliations:** Department of Neurosurgery, Alfred Health, 55 Commercial Rd, Melbourne, VIC 3004, Australia; NeuroSpine Clinic, Prince of Wales Private Hospital, Suite 7, level 7/346 Barker St., Randwick, NSW 2031, Australia; Department of Neurosurgery, University of New South Wales, Barker Street, Randwick, NSW 2031, Australia; One Brain and Spine, St Vincent’s Private Hospital, Suite 1, level 10, East Tower/59 Victoria Parade, Fitzroy, VIC 3065, Australia

**Keywords:** endoscopic spine surgery, multi-portal endoscopy, robotic assistance, intradural, arachnoid cyst, thoracic spine

## Abstract

Endoscopic treatment of spinal intradural lesions is technically demanding because bimanual manipulation, irrigation control, neural protection, and dural closure are constrained. A 44-year-old woman with myelopathy from a dorsal T4–T10 intradural arachnoid cyst underwent endoscopic fenestration at T4/5. The hybrid multi-portal technique combined unilateral biportal-style exposure, a uniportal working-channel endoscope, intraoperative navigation, and robotic-arm camera stabilization. An accessory portal enabled bimanual intradural manipulation. Irrigation pressure was reduced after durotomy, and the dura was closed with sutures and clips reinforced by sealants. Day 1 magnetic resonance imaging confirmed cord decompression, and lower-limb sensory and bladder symptoms had resolved at 6 weeks. To our knowledge, this is the first reported navigated robotic-arm-assisted hybrid multi-portal endoscopic fenestration of a thoracic intradural arachnoid cyst. This single case demonstrates technical feasibility but not superiority over open or microscopic surgery.

## Introduction

Symptomatic spinal intradural arachnoid cysts are traditionally treated through open or microscopic exposure when conservative management is not appropriate. Endoscopic treatment has been reported for selected arachnoid cysts and other intradural pathologies, but the evidence remains limited to small series, technical notes and case reports [1–8].

Intradural endoscopic surgery is difficult for a different reason to routine extradural degenerative work. The surgeon must control the cyst wall, neural elements, and dura in a confined irrigated field. Uniportal full-endoscopic surgery provides close-up visualization but restricts independent two-handed manipulation. The unilateral biportal endoscopic (UBE) approach improves instrument freedom, but irrigation-pressure control becomes important once the dura is opened. In both techniques, dural closure remains difficult being single-handed. Multi-portal endoscopy may address part of this problem by combining stable endoscopic visualization with an independent accessory working corridor, allowing bimanual manipulation [9, 10].

We report a thoracic intradural arachnoid cyst treated using a hybrid multi-portal technique. The approach combined UBE-style exposure, a uniportal working-channel endoscope, intraoperative navigation, and robotic arm stabilization of the endoscope. In this report, we describe the technique and the early clinical and radiological outcomes.

## Case report

A 44-year-old Caucasian woman presented with a 6-month history of bilateral lower limb paraesthesiae, feet numbness, and occasional urinary incontinence, on a background of otherwise good health. Full-spine magnetic resonance imaging (MRI) demonstrated a large posterior thoracic arachnoid cyst extending from T4 to T10, causing anterior displacement and compression of the thoracic cord (Fig. 1).

**Figure 1:**
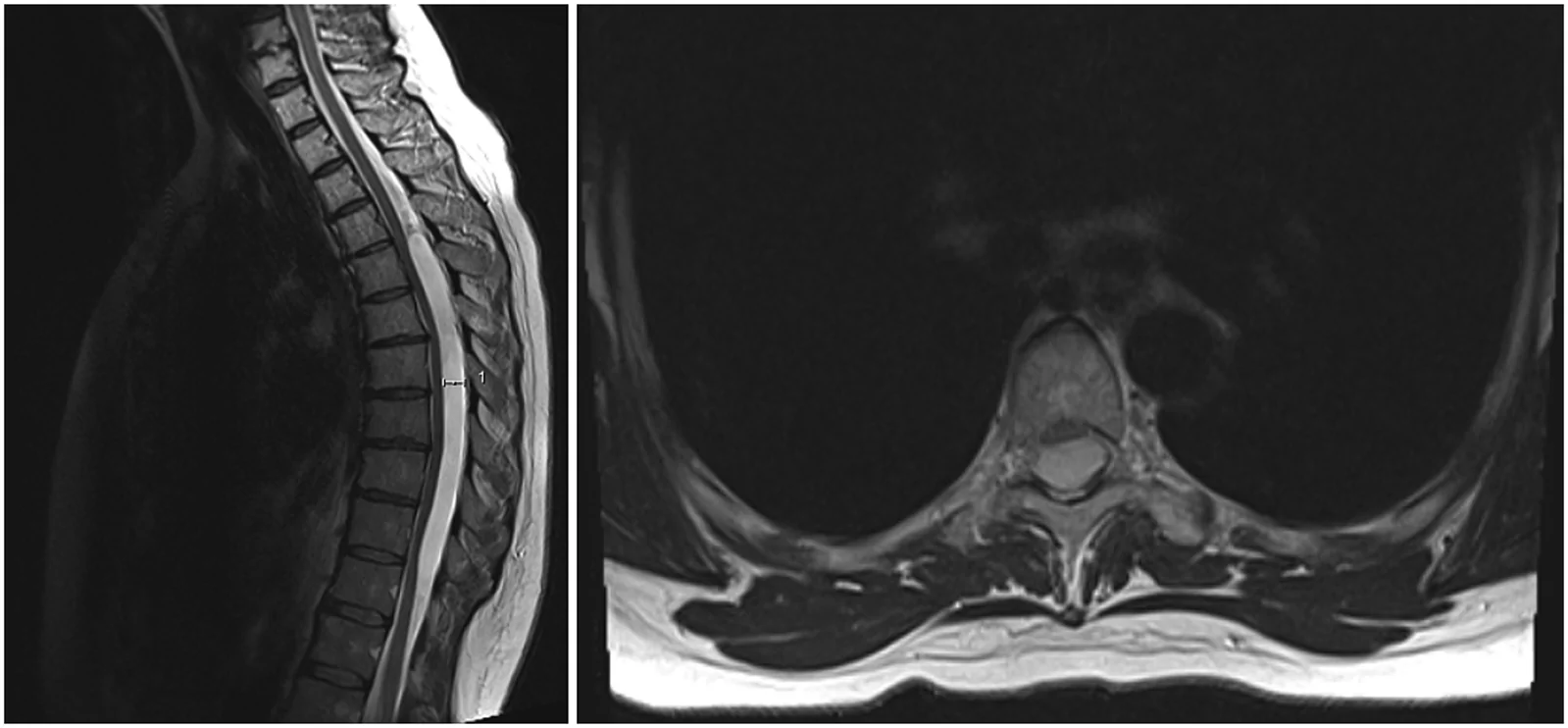
Preoperative MRI. (A) Sagittal and (B) axial T2-weighted images show a dorsal thoracic arachnoid cyst with anterior displacement and compression of the cord.

She was consented for endoscopic fenestration of the thoracic intradural arachnoid cyst at the T4/5 level at St Vincent’s Private Hospital, Melbourne.

## Operative technique

On the day of surgery, the patient underwent general anesthesia and was positioned prone on a counter-levered radiolucent Trumpf table. Intraoperative navigation was used after securing a spinous process clamp at T7/8 and obtaining 3D intraoperative images with the mobile imaging robot Loop-X (Brainlab AG, Munich, Germany). The intraoperative computed tomography (CT) was merged with the pre-operative MRI, and the relevant vertebral levels were segmented. The planned portals were then marked and established using Matrix Endoscopy equipment, with the instruments triangulated toward T4/5 (Fig. 2).

**Figure 2:**
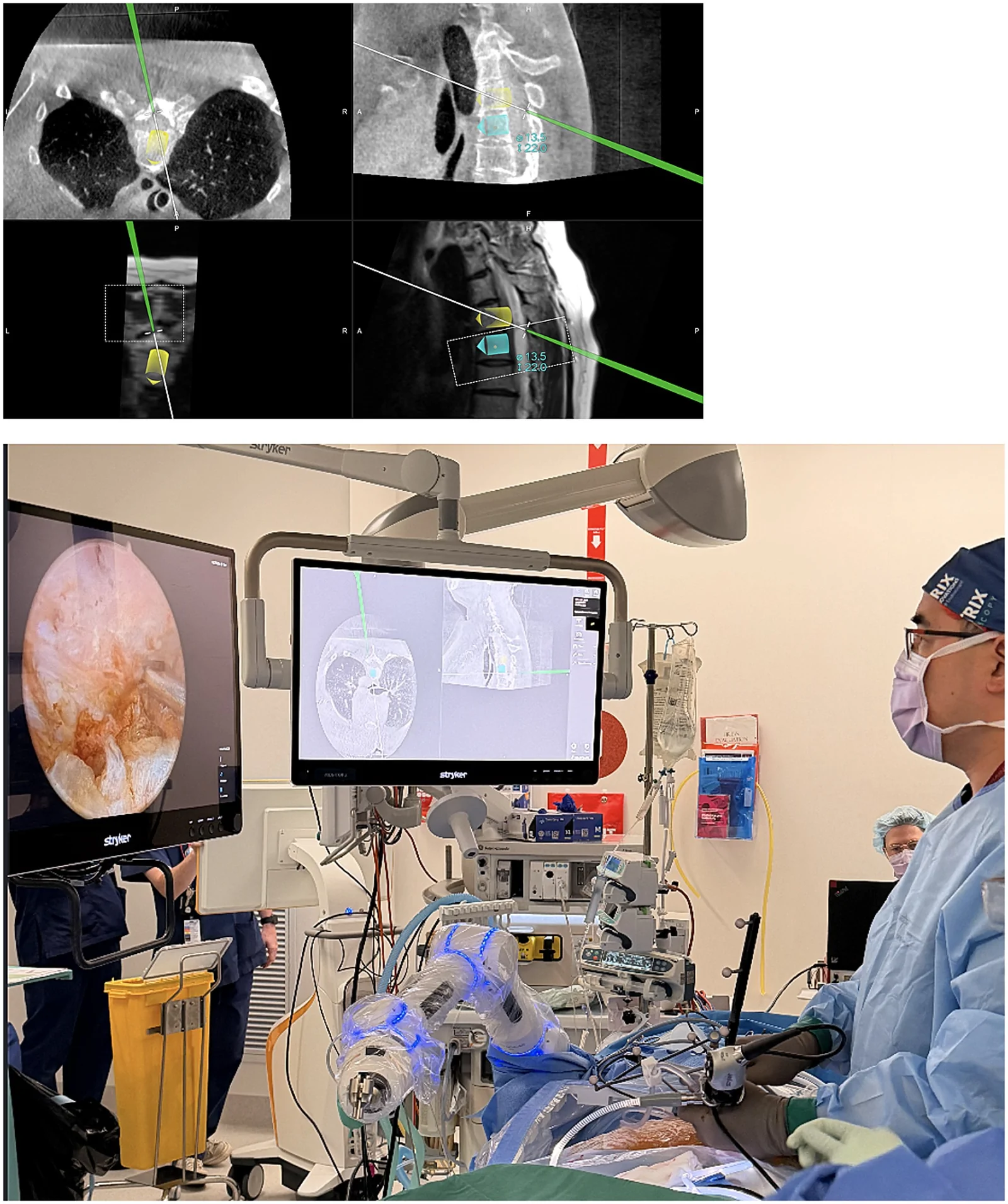
Intraoperative level check and navigation. (A) Loop-X intraoperative CT merged with preoperative MRI shows the planned navigated trajectory at T4/5. (B) Operating-room view of the navigation display, endoscopic display, and robotic arm.

The hybrid multi-portal construct consisted of a uniportal working-channel endoscope and an accessory working portal. The endoscope allowed instrument passage through its working channel. The accessory portal allowed a second instrument to be introduced independently, enabling bimanual surgery. The endoscopic camera was held by the navigated Brainlab Cirq robotic arm, which maintained a stable endoscopic trajectory during the intradural phase whilst minimizing the risk of inadvertent “plunging” (Fig. 3).

**Figure 3:**
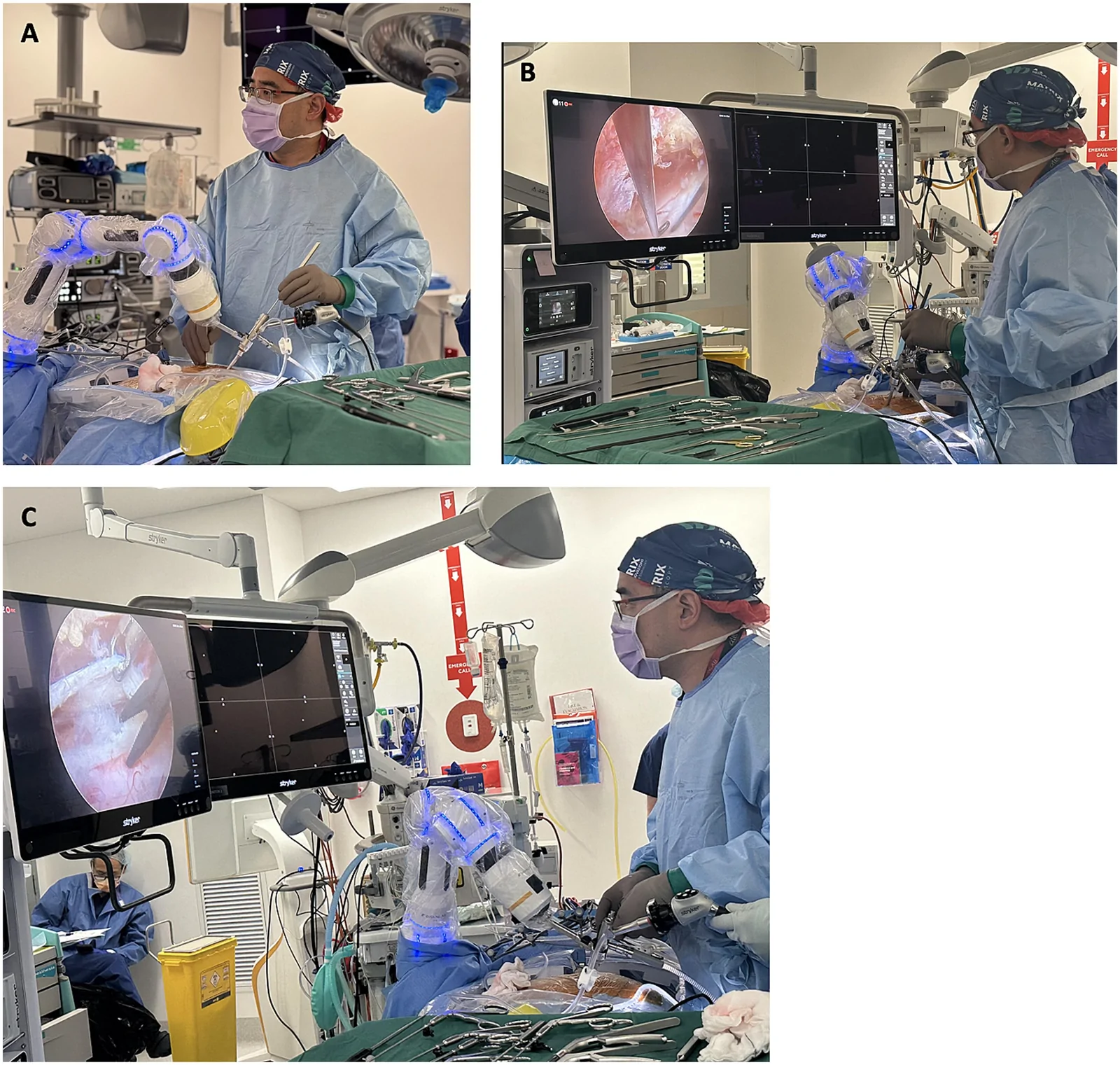
Hybrid multi-portal bimanual endoscopic fenestration. (A) The navigated robotic arm stabilizes the endoscope. (B) The endoscopic display shows dural opening under direct visualization. (C) The display shows bimanual intradural work while the robotic arm maintains the endoscopic trajectory.

The surgical exposure began as a standard UBE-style decompression. A right-sided unilateral laminectomy was performed for bilateral decompression from T3/4 to T5/6 using 5 and 3 mm diamond drills and spinal curettes. Irrigation pressure was set at 60 cmH_2_O during the extradural decompression, reduced to 40 cmH_2_O once the dura was exposed and then reduced further to 15 cmH_2_O once the dura was opened.

The dura was sharply opened in the midline under endoscopic visualization using a bimanual technique. The intradural arachnoid cyst was fenestrated and partially resected using microscissors, pituitary rongeurs, and fine forceps. The dura was closed using 5–0 Prolene sutures and dural clips, then reinforced with onlay TachoSil and Tisseel. The procedure can be viewed in Supplementary Video 1.

## Postoperative course

Histopathological examination of the resected material confirmed an arachnoid cyst. Day 1 postoperative MRI showed reduction in the size of the dorsal arachnoid cyst, increased flow void within the cyst, and decompression of the thoracic cord (Fig. 4). The patient was discharged home on postoperative Day 2.

**Figure 4:**
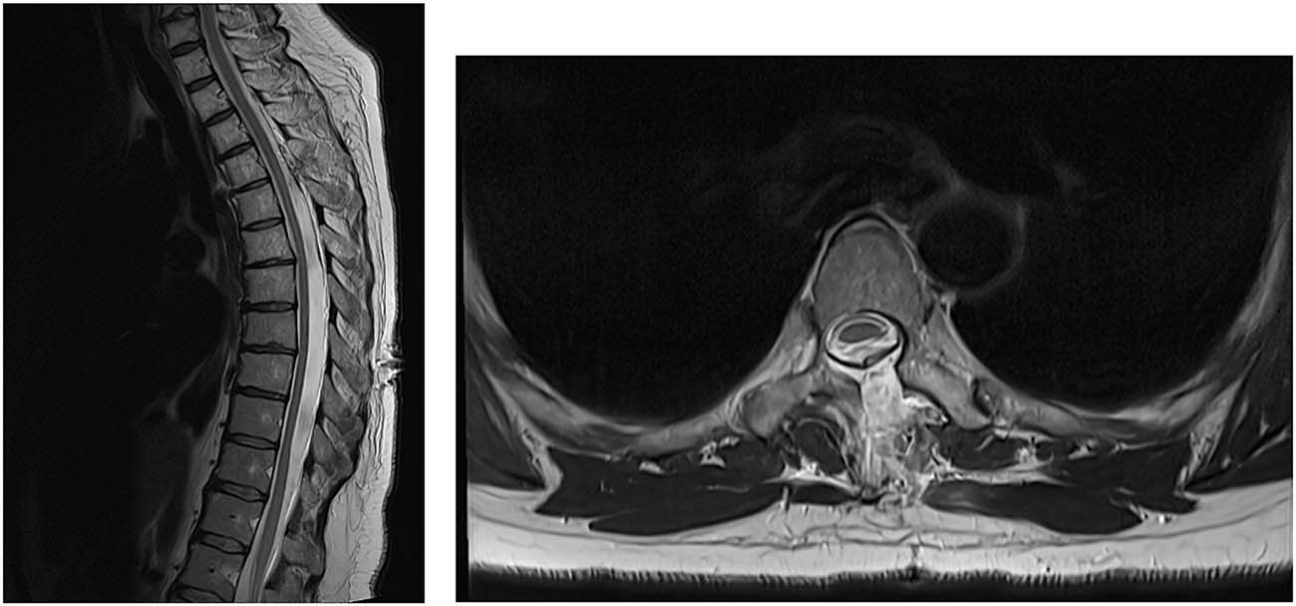
Day 1 postoperative MRI. (A) Sagittal and (B) axial T2-weighted images show reduction in the dorsal arachnoid cyst, increased flow void within the cyst, and decompression of the thoracic cord.

At 6-week review, she was doing well, with resolution of her pre-operative lower limb and bladder symptoms. The surgical wounds are shown in Fig. 5. She is planned to undergo further MRI at 6 months postoperatively.

**Figure 5:**
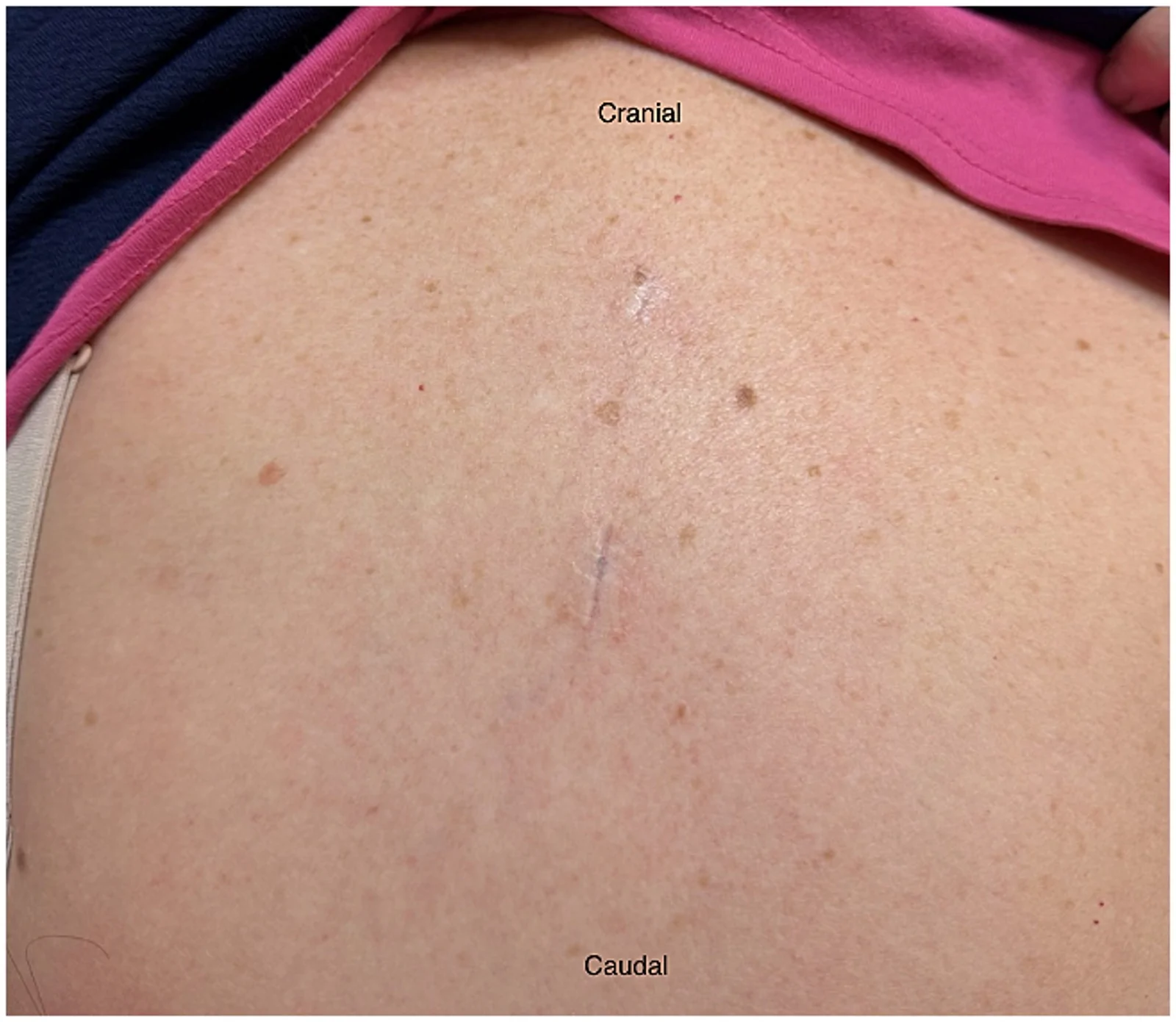
Surgical wounds at 6 weeks. Two small linear upper-thoracic incisions have healed.

## Discussion

Endoscopic techniques have been described for spinal intradural arachnoid cysts, tethered cord, intradural extramedullary tumors and other intradural lesions [1–8]. Recent reports also describe UBE resection of thoracic and cervical intradural meningiomas [5, 6]. The specific contribution of the present case is the combination of UBE-style exposure, a uniportal working-channel endoscope, navigation, and robotic camera stabilization to support bimanual intradural fenestration of a thoracic arachnoid cyst.

The case extends the current endoscopic spine surgery envelope beyond its usual extradural degenerative indications and into selected thoracic intradural pathology. Intradural surgery places different demands on the operative corridor. The surgeon needs stable visualization, fine two-handed tissue control, neural protection, careful irrigation-pressure management, and a reliable dural closure. In this setting, the hybrid construct may combine useful features of the two major endoscopic lineages: close-up visualization and working-channel access from uniportal endoscopy, with triangulation and instrument freedom from biportal surgery.

The robotic arm was useful because it stabilized the camera and reduced reliance on a human camera holder during the intradural phase. This is a practical point rather than a claim of technological superiority. A stable endoscopic trajectory makes bimanual work easier when small movements matter. It also reduces the chance that the field is lost during cyst wall manipulation or dural closure.

The case also highlights the limits of this approach. It is a single case with short follow-up. It does not prove superiority over open or microscopic intradural surgery. The technique requires expertise in endoscopic decompression, careful corridor planning, bleeding control, irrigation-pressure discipline, and dural closure. Once the dura is opened, fluid pressure becomes a central safety issue rather than a background technical variable.

Further experience is needed to define suitable lesions and to decide whether this method can be standardized and taught. At present, the most appropriate role is likely to be selected intradural cysts or small extramedullary lesions in expert hands, where the planned corridor is direct and where bimanual endoscopic control adds a real advantage.

## Conclusion

This case demonstrates the technical feasibility of navigated robotic-arm-assisted hybrid multi-portal endoscopic fenestration of a thoracic intradural arachnoid cyst. The key technical features were stable endoscopic visualization, bimanual intradural manipulation, deliberate irrigation-pressure reduction after dural opening, and reinforced dural closure. The approach does not establish superiority over open or microscopic surgery, but it provides a practical framework for further study of selected intradural endoscopic procedures.

## Supplementary Material

Supplementary_material_rjag998

## References

[1] Endo T, Takahashi T, Jokura H et al. Surgical treatment of spinal intradural arachnoid cysts using endoscopy. J Neurosurg Spine 2010;12:641–6. 10.3171/2009.12.SPINE09577 20515350

[2] Endo T, Tominaga T. Use of an endoscope for spinal intradural pathology. J Spine Surg 2020;6:495–501. 10.21037/jss.2020.01.06 PMC7340816 32656387

[3] Papadimitriou K, Cossu G, Maduri R et al. Endoscopic treatment of spinal arachnoid cysts. Heliyon 2021;7:e06736. 10.1016/j.heliyon.2021.e06736 PMC8050863 33889785

[4] Tredan DAM, Mobbs RJ, Ma A et al. Uniportal endoscopic spine surgery for removal of a thoracic arachnoid cyst: a technical note. J Clin Neurosci 2025;140:111530. 10.1016/j.jocn.2025.111530 40803082

[5] Peng W, Zhuang Y, Cui W et al. Unilateral Biportal endoscopy for the resection of thoracic intradural extramedullary Tumors: technique case report and literature review. Int Med Case Rep J 2024;17:301–9. 10.2147/IMCRJ.S444226 PMC11015842 38618188

[6] Jung SB, Kim N. Biportal endoscopic resection of intradural meningioma in the cervical spine: a case report. Int J Spine Surg 2024;18:611–6. 10.14444/8645 PMC11616400 39242179

[7] Dolas I, Unal TC, Gulsever CI et al. Full-endoscopic spine surgery for intradural pathologies: a systematic review of innovations, challenges, and future prospects. Brain Spine 2025;5:104392. 10.1016/j.bas.2025.104392 PMC12375244 40862227

[8] Hagel V, Isseldyk FV. Full-endoscopic resection of a lumbar intradural tumor (schwannoma): video case report and description of the surgical technique. Neurospine 2024;21:1096–9. 10.14245/ns.2449080.540 PMC11744534 39765247

[9] Yang L, Zhou L, Wang G et al. Unilateral bi/multi-portal endoscopy for the treatment of complicated lumbar degenerative diseases with utilization of uniaxial spinal endoscope, instead of arthroscope: technique note and clinical results. Clin Interv Aging 2023;18:1295–308. 10.2147/CIA.S417462 PMC10423581 37581123

[10] Kim JE, Choi DJ, Park EJJ et al. Biportal endoscopic spinal surgery for lumbar spinal stenosis. Asian Spine J 2019;13:334–42. 10.31616/asj.2018.0210 PMC6454273 30959588

