# Supplementary material for: Navigated robotic-arm-assisted hybrid multi-portal endoscopic fenestration of a thoracic intradural arachnoid cyst: a technical case report
Source: J Surg Case Rep. 2026 Nov 4;2026(11):rjag998. doi: 10.1093/jscr/rjag998 (PMC13731194; doi:10.1093/jscr/rjag998)
Supplement: Supplementary file 1 [file supplementary_material_rjag998.zip › Supplementary_Caption.docx]

Supplementary video legend

Supplementary Video 1. Navigated robotic-arm-assisted hybrid multi-portal endoscopic fenestration of a thoracic intradural arachnoid cyst.

The video demonstrates biportal-style exposure; staged irrigation-pressure reduction; bimanual midline durotomy; cyst fenestration and partial resection with microscissors, pituitary rongeurs and fine forceps; and dural closure with 5‑0 polypropylene sutures and dural clips reinforced with TachoSil and Tisseel.
